# Green-synthesized zinc oxide nanoparticles by *Enterobacter sp.*: unveiling characterization, antimicrobial potency, and alleviation of copper stress in *Vicia faba* (L.) plants

**DOI:** 10.1186/s12870-024-05150-0

**Published:** 2024-05-30

**Authors:** Sobhy E. Elsilk, Rania A. El-Shenody, Salsabil S. Afifi, Walaa A. Abo-Shanab

**Affiliations:** Botany and Microbiology Department, Faculty of Science, Tanta University, Tanta, 31527 Egypt

**Keywords:** *Enterobacter sp*., ZnO NPs, Antimicrobial, Antioxidants, Copper stress, *Vicia faba*

## Abstract

**Background:**

The biosynthesis of zinc oxide nanoparticles (ZnO NPs) using *Enterobacter sp.* and the evaluation of their antimicrobial and copper stress (Cu^+ 2^)-reducing capabilities in *Vicia faba* (L.) plants. The green-synthesized ZnO NPs were validated using X-ray powder diffraction (XRD); Fourier transformed infrared (FTIR), Ultraviolet-Visible spectroscopy (UV-Vis), Transmission electron microscope (TEM) and scanning electron microscopy (SEM) techniques. ZnO NPs could serve as an improved bactericidal agent for various biological applications. as well as these nanoparticles used in alleviating the hazardous effects of copper stress on the morphological and physiological traits of 21-day-old *Vicia faba* (L.) plants.

**Results:**

The results revealed that different concentrations of ZnO NPs (250, 500, or 1000 mg L^-1^) significantly alleviated the toxic effects of copper stress (100 mM CuSO_4_) and increased the growth parameters, photosynthetic efficiency (Fv/Fm), and pigments (Chlorophyll a and b) contents in Cu-stressed *Vicia faba* (L.) seedlings. Furthermore, applying high concentration of ZnO NPs (1000 mg L^-1^) was the best dose in maintaining the levels of antioxidant enzymes (CAT, SOD, and POX), total soluble carbohydrates, total soluble proteins, phenolic and flavonoid in all Cu-stressed *Vicia faba* (L.) seedlings. Additionally, contents of Malondialdehyde (MDA) and hydrogen peroxide (H_2_O_2_) were significantly suppressed in response to high concentrations of ZnO NPs (1000 mg L^-1^) in all Cu-stressed *Vicia faba* (L.) seedlings. Also, it demonstrates strong antibacterial action (0.9 mg/ml) against various pathogenic microorganisms.

**Conclusions:**

The ZnO NPs produced in this study demonstrated the potential to enhance plant detoxification and tolerance mechanisms, enabling plants to better cope with environmental stress. Furthermore, these nanoparticles could serve as an improved bactericidal agent for various biological applications.

**Supplementary Information:**

The online version contains supplementary material available at 10.1186/s12870-024-05150-0.

## Background

Zinc oxide (ZnO) has recently emerged as a significant chemical compound, garnering widespread interest due to its versatile applications across various fields [1, 2]. The antimicrobial properties inherent in nanomaterials have positioned them as nano-antibiotics, marking a notable advancement in their functionality [3]. The extensive integration of nanomaterials into consumer products spanning nutrition, health, supply chains, space, chemicals, and cosmetics necessitates a conscientious approach to their production with environmental considerations at the forefront [4]. The biosynthesis of nanoparticles by plants and microbes opens avenues for applications in biomedicine. This approach facilitates the large-scale production of ZnO NPs without introducing additional contaminants, thereby addressing environmental concerns [5]. Notably, nanoparticles created through a biomimetic method exhibit heightened catalytic activity while minimizing the reliance on costly and toxic chemicals. This underscores the potential for sustainable and efficient production methods in the realm of nanomaterials.

The synthesis of nanomaterials through green routes has garnered considerable attention due to its eco-friendly and sustainable nature. Among these, ZnO NPs have emerged as promising candidates with diverse applications, ranging from antimicrobial to stress-alleviating plant agents. The green method of synthesizing nanoparticles using many bacterial species involves taking various safety measures, such as closely monitoring the microbial broth during the whole process to prevent infection [4]. *Bacillus licheniformis* (*B. licheniformis*) produced ZnO nanoflowers using a green method and reduced the color Methylene blue [6]. It is assumed that the greater vacancy of oxygen in the nanoparticles that have been synthesized imparts an attribute of improved photocatalytic capacity and produces active species by consumption of light which decreases organic debris and could be used as an efficient bioremediation tool because these nanoflowers demonstrated enhanced photocatalytic activity [4]. The diameter and height of the nanoflowers produced using *B. licheniformis* reached 40 nm and 400 nm, respectively [7].

In addition to its ability to metabolize hydrophobic chemicals and endure harsh environments, *Rhodococcus* also plays a role in their biodegradation [8]. *Rh. pyridinivorans* and zinc sulfate were used as a substrate to create spherical nanoparticles (NPs), with an XRD and FE-SEM study confirming their size ranging from 100 to 130 nm [9]. A confirmation of the existence of amide II stretching band, enol of 1-3-di ketone, hydroxyaryl ketone, alkane, nuclear benzene band, amide 1 bending band, monosubstituted alkyne, β-lactone, amines salt, and phosphorus compound was also provided by FTIR examination [5]. ZnO was used as a substrate by *Aeromonas hydrophila* (*A. hydrophilla*) to create ZnO NP. XRD examination verified the size range of the produced NPs, which ranged from 42 to 64 nm and had a variety of morphologies, including spherical and oval [10].

Additionally, Singh et al. [11] examined the antioxidant capacity of ZnO NPs and *Pseudomonas aeruginosa (P. aeruginosa)* rhamnolipid stabilized NPs. They discovered that rhamnolipid stabilizes ZnO NPs since micelle gathers on the outer layer of carboxymethyl cellulose are difficult to develop. Because of its long carbon chain, it functions as a better capping agent. Through TEM and XRD investigation, it demonstrated the synthesis of spherical-shaped NPs with nano sizes of 27–81 nm [11]. TEM and XRD investigation demonstrated that Lactobacillus “Bacillaceae” formed hexagonal ZnO NPs with a nano-size of 5–15 nm [12].

Probiotic lactic acid bacteria (LAB), one of the microorganisms used to synthesize ZnO NP, have drawn a lot of attention because of their advantageous and non-pathogenic characteristics. Gram-positive LAB is an aerobic bacterium that belongs to both function groups along with the biostructures group and has thick walls of cells [13]. To aid in the creation of ZnO NPs, these functional groups bind to the metal ions. Moreover, LAB secretes a variety of enzymes that assist in stabilizing and reducing ZnO NPs. Therefore, employing either cell-biomass or cell-free supernatant, different studies have been conducted to ascertain probiotic LAB’s effectiveness in regulating the synthesis of ZnO NPs [1]. ZnO NP powder’s antimicrobial activity against fungi, *Escherichia coli (E. coli)*, and *Staphylococcus aureus* (*S. aureus*) was qualitatively assessed in culture medium [14]. It was thought that the metal oxide particles identified as active oxygen species could be the primary source for their antibacterial effects. NPs can be used as fungicides due to their mycoses [15]. Nevertheless, the impacts of nanoparticles against *S. aureus* and *E. coli* as well as on fungal infections, are currently being evaluated in a few studies [16]. The antibacterial abilities of nano-TiO2 and oxidants of other nanomaterials, such as CdO and ZnO, have been reported, in addition to the bactericidal characteristics of nanosilver [17].

The effects of zinc on plant development and proliferation have been the subject of numerous investigations. Since it is the sole metal found in all six types of enzymes, ligases, hydrolases, lyases, isomerases, oxidoreductases, and transferases all contain it [18]. Plant functions, including photosynthesis and the creation of DNA and RNA, depend on zinc in one way or another [19]. Zinc amendment is so crucial and utilized to promote the production of cereals, vegetables, and fodder [20]. Zinc is necessary for plants to make pollen, metabolize carbohydrates, synthesize enzymes, preserve the integrity of cell membranes, and control the synthesis of auxin [18]. Additionally, it controls the expression of genes crucial for resistance to environmental stressors like intense light or temperatures. Plants that are zinc deficient exhibit aberrant growth of their structures. The symptoms of acute deficiency include sterility, reduced leaf area, chlorosis of the leaves, and delayed growth [19].

However, ZnO NPs regarded to be one of the most significant nanomaterials and are frequently utilized as nanofertilizers, particularly in locations with a Zn shortage, to promote plant development and growth [21]. According to [22] adding ZnO NP may help with nutritional deficiencies and increase agricultural yields. Due to ZnO NPs’ extensive use in the agricultural industry, recent research has demonstrated a favorable effect on plant development and physiology [23]. Additionally, several studies investigated how adding ZnO NPs to soil-amended areas enhanced biomass formation and decreased the harmful effects of heavy metal absorption in various plant species [24].

The capacity of ZnO NPs to move across soils and interact with soil constituents is one of its most crucial characteristics. According to [25–28], and others, the behavior of released Zn ions is the most significant factor in plant bioavailability in an acidic environment where Zn ONPs can form big aggregates and dissolve easily (partially or totally). The dissolution is significantly reduced in more alkaline conditions, and in some cases, ZnO NPs can be transported more readily than ionic Zn even though both forms may attach to different soil constituents similarly [29]. Zn ONPs have been demonstrated to enhance seed germination, growth [30], photosynthesis [31], antioxidant enzyme activity [32], chlorophyll production [33], proteins, oil, and seeds [34, 35], as well as micronutrient absorption [33]. Additionally, it was discovered that they mitigated abiotic stressors, such as temperature [36], heavy metals [37], salt [38], and drought [39].

Additionally, the current investigation seeks to assess *E. sp.*‘s biosynthesized ZnO NP which have good biological uses and are very affordable and less hazardous. Furthermore, the ZnO NPs mitigate the harmful effects of copper stress on *V. faba* (L.) and can used as a stress-reduction tool for crops cultivated in Cu-contaminated areas to promote crop development and production. The assessment was conducted on the morphological and physiological traits of 21-day-old *Vicia faba* (L.) seedlings in response to ZnO NPs.

## Materials and methods

### Isolation and identification of bacteria used in

On a Man, Rogosa, and Sharpe (MRS) medium, bacteria used to synthesize ZnO NPs were separated from yogurt. Following purification, the isolated strain was identified using 16s rRNA and validated by biochemical testing.

### Preparation of supernatants

An inoculum of the discovered bacterial strain *E. sp.* cultivated recently and used to prepare, sanitize, and inoculate MRS broth. For 48 h, the culture flasks were incubated at 35 °C. Following the incubation, the cultures were centrifuged at 6000 rpm to extract the supernatants, which were then utilized in additional studies.

### Synthesis of ZnO NPs

After mixing 50 ml of sodium hydroxide and zinc sulfate (0.1 M and 0.4 M, respectively) with 50 mL of supernatant, the mixture was agitated briskly and heated to 40 °C for one hour. Then the flask was placed in a microwave oven for two minutes, and cooled for one hour to allow the nanoparticles to settle. The white color deposition at the flask bottoms served as evidence that nanoparticles were forming. The nanoparticles were transported to the centrifuge tubes and distilled water was added. Centrifugation was carried out repeatedly for ten minutes at 3000 rpm. After gathering the pellet in a tiny plate, it dried in an oven set at 40 °C until it was completely dry, and powdered ZnO NPs were produced [40].

### Characterization of ZnO NPs

#### Ultraviolet-visible spectroscopy (UV-Vis spectroscopy)

Using a Cecil model 9200 Ultraviolet-visible spectrophotometer operated at a resolution of 1 nm, the UV-Vis spectrum of solutions was measured to characterize the reduction of Zn through the supernatant of the examined bacteria in the solution as well as the resulting formation of ZnO NPs. The aqueous component (2 mL) was sampled, and the UV-Vis spectrum of solutions was determined.

#### X-Ray diffraction spectroscopy (XDR)

ZnO NPs’ particle size, shape variation, and spatial distribution were all assessed using X-ray diffraction spectroscopy (XRD). Evaluation investigations were conducted with the white crystalline ZnO NPs. Using a diffractometer, one-line reactor, and a Phillips PW 1729/40 generator, the powder sample was examined across an extensive variety of Bragg angles (20–80 ˚C) with Cu Kα radiation (λ = 1.5405 A).

#### Transmission Electron Microscopy (TEM)

The test bacteria’s culture supernatants produced ZnO NPs, which were utilized for transmission electron microscopy (TEM; Joel, 100SX, Japan with AMT digital camera). After ultrasonically dispersing each specimen to isolate the individual particles, a few drops of the solution were placed onto copper grids covered with holes in carbon and allowed to dry under an infrared lamp. Photographs and observations were taken of the ZnO NP film.

#### Fourier-transformed infrared analysis (FTIR)

ZnO NPs with a functional group range from 4000 to 400 cm^− 1^ were analyzed using FT-IR. Using a hydraulic press, pellets produced after ZnO NPs were implanted in the KBr matrix. At a resolution of 4 cm^− 1^, samples were scanned in the 400–4000 cm^− 1^ frequency range to capture the FTIR spectra.

### Inhibitory activity

The test microorganisms *E. coli, S. typhimurium, K. pneumoniae*, and *C. albicans* were procured from the Bacteriology Unit’s culture collection at Tanta University’s Botany Department, Faculty of Science. To grow the test bacterium and the yeast that resembles a fungus, Sabouraud’s broth and nutrient-rich agar are utilized by *C. albicans*. Using plates sown with the bacteria that examined and inoculum fungus, the cut plug technique was used to investigate the antibacterial spectrum of the produced ZnO NPs [41]. In nutrient and sabouraud agar, night cultures of the bacterial and *C. albicans* indicators were grown, with inoculums diluted to 1 × 10^5^ cfu mL ^− 1^. The wells with a diameter of 5 mm were filled with 200 µL of ZnO NPs that had been previously created by the strain of bacteria that was being studied, along with 200 µL of 1 × 10^5^ cfu mL^− 1^ for the fungal and bacterial indicators. Following a 24-hour incubation period at 30 °C, the diameters of the regions of inhibition on the plates were measured to determine the inhibitory effects and the data obtained compared with standard antibiotics tetracycline.

### Plant material

The faba bean seeds (Vicia faba cv. Giza 3) were obtained from the Sakha Research Station, Kafr El-Sheikh, Agriculture Research Center, Department of Agronomy, Egypt. Before use, the seeds were disinfected by washing them in 1% NaClO for two minutes and then rinsing them three times in sterile distilled water.

### Preliminary experiments

Faba bean seeds of uniform size and shape were soaked in distilled water for 10 h. Six of the soaked seeds were sown in each plastic pot; *n* = 15 (10 cm diameter and 15 cm depth) containing one Kg of clay-sandy soil (2:1 w/w). The sawn seeds were watered with every two days with different concentrations of CuSO_4_ (50, 100, 150, 200, and 250 mM) to 80% field capacity. The seedlings were left to grow in the greenhouse for 7 days then the germination percentages were detected to determine the lethal and sub-lethal concentrations of CuSO_4_.We chose 100 mM CuSO_4_ as the sub-lethal dose that was found to have the most negative impacts based on the results of our initial screening. Plants showed signs of chlorosis before symptoms developed and leaf necrosis patches appeared. Moreover, different concentrations of ZnO NPs (250, 500, and 1000 mg L^− 1^) were prepared and applied according to [42].

### Main experiment and growth conditions

During the growing season (October 2022), seeds of faba beans of uniform size and shape were soaked in distilled water for 10 h before sowing. After that, seeds were divided into ten groups of three replicates (*n* = 3; 30 pots) and ten seeds were sown in each plastic pot (25 cm diameter and 30 cm depth) filled with 15 kg clay-sandy soil (2:1w/w) and placed in a randomized complete block design as follows; the first group was irrigated with distilled water (control), the 2nd group was irrigated with CuSO_4_ solution (100 mM), the 3rd group was irrigated with bacterial suspension, 4th, 5th and 6th groups were irrigated with different concentrations of ZnO NPs solutions (250, 500, and 1000 mg L^-1^, respectively) moreover, the 7th group was irrigated with bacterial suspension + 100 mM CuSO_4_ solution while 8th, 9th and 10th groups were irrigated with different concentrations of ZnO NPs solutions (250 mg L^-1^ + 100 mM CuSO_4_, 500 mg L^-1^ + 100 mM CuSO_4_, and 1000 mg L^-1^ + 100 mM CuSO_4_, respectively) till 80% field capacity. Then the seeds were left to grow to represent the seedling stage. Under the conditions of 11 h light and 13 h dark, at 28 °C ± 2 and 16 °C ± 2, respectively with 62% relative humidity, the sown seeds were left in the greenhouse for 7 days to germinate while they were watered with tap water every two days for the first week (before seedling emergence). On the 7th and ^14^ th days, all the groups were watered with previuos prepared solutions except the control which was watered with water. Thereafter, all seedlings were watered with water twice a week then The 21-day-old *Vicia faba* (L.) seedlings were harvested, washed with water to get rid of soil particles, and separated into roots, shoots and leaves. The fresh mass (FM) of all samples was determined. For at least 72 h, the samples were dried at 50 °C in the oven to a constant weight for determination of dry mass (DM). The growth criteria {shoot height, leaf area, shoot fresh mass (FM), root fresh mass (FM), shoot dry mass (DM), and root dry mass (DM)} were determined.

### Photochemical measurements

According to [43] for chlorophylls and carotenoids, as accepted by [44] the photosynthetic pigments chlorophyll a (Chl a), chlorophyll b (Chl b), and carotenoids (Carot.) identified in the leaves of the 21-day-old seedlings. A chlorophyll fluorometer (OS-30 p) was used to quantify the fluorescence parameters by determining the maximum efficiency of a photosystem PSII (FV/Fm) of leaves that were acclimated to darkness (Hudson, NH 03051 USA) [45].

### Physiological analysis

MDA concentration was determined using the [46] technique, and the extinction coefficient (155 mM^− 1^ cm^− 1^) was used to quantify the lipid peroxidation level. The hydrogen peroxide (H_2_O_2_) was calculated using the method described by [47]. Superoxide dismutase [EC1.15.1.1] (SOD) activity calculated by [48] Catalase[EC1.11.1.6] (CAT) and peroxidase [EC1.11.1.7] (POX) activities were measured by [49] and reported in units of U min^− 1^ mg^− 1^ protein. According to the [49] method, the total soluble protein content of *V. faba* leaves is quantitatively assessed. The phenol-sulfuric acid method was used by [50] to calculate the total quantity of soluble carbohydrates. The total concentration of phenolic and flavonoid content has been determined according to the previous study [51].

### Statistical analysis

All measurements were performed in three biological replicates, with each treatment replicated three times (*n* = 3), with data expressed as mean ± standard deviation (SD). One-way analysis of variance (ANOVA) was conducted, followed by Duncan’s multiple range test for post-hoc comparisons among the treatment groups. Statistical differences were determined at a significance level of 0.001. A heat map of Pearson’s correlation coefficients was computed among variables using the SPSS software (version 23) to interpret the relationships between the treatments and measured parameters. The use of multivariate classification, also known as cluster analysis, was employed to improve the processes of interpretation and determine the degree of similarity between many treatments.

## Results

**Fig. 1:**
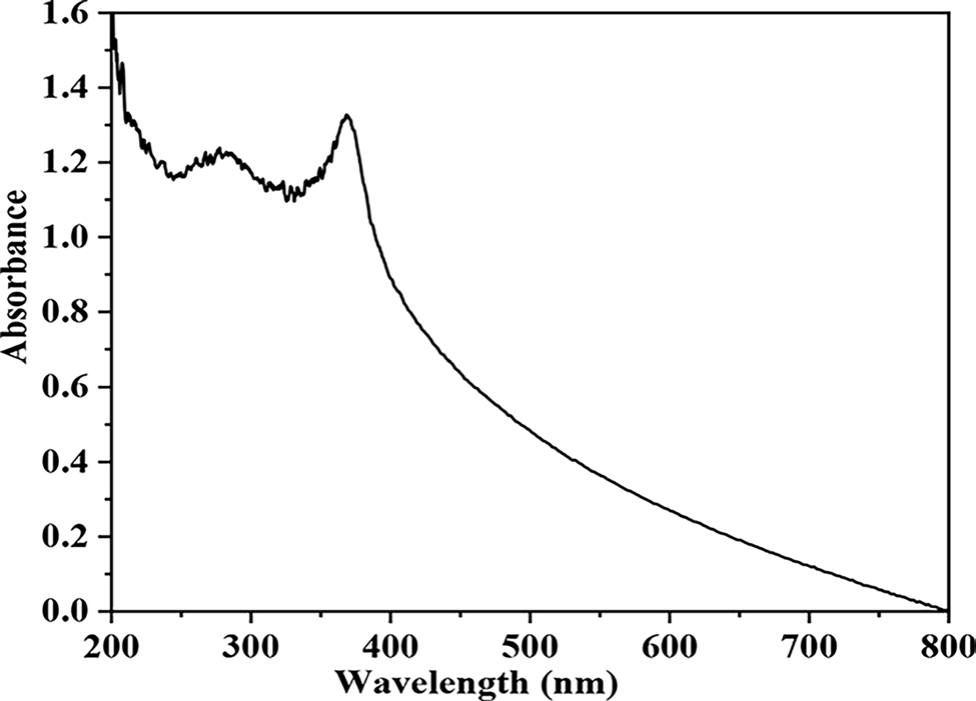
UV–visible spectrum of ZnO NPs synthesized using bacterial extract of *Enterobacter sp*

**Fig. 2:**
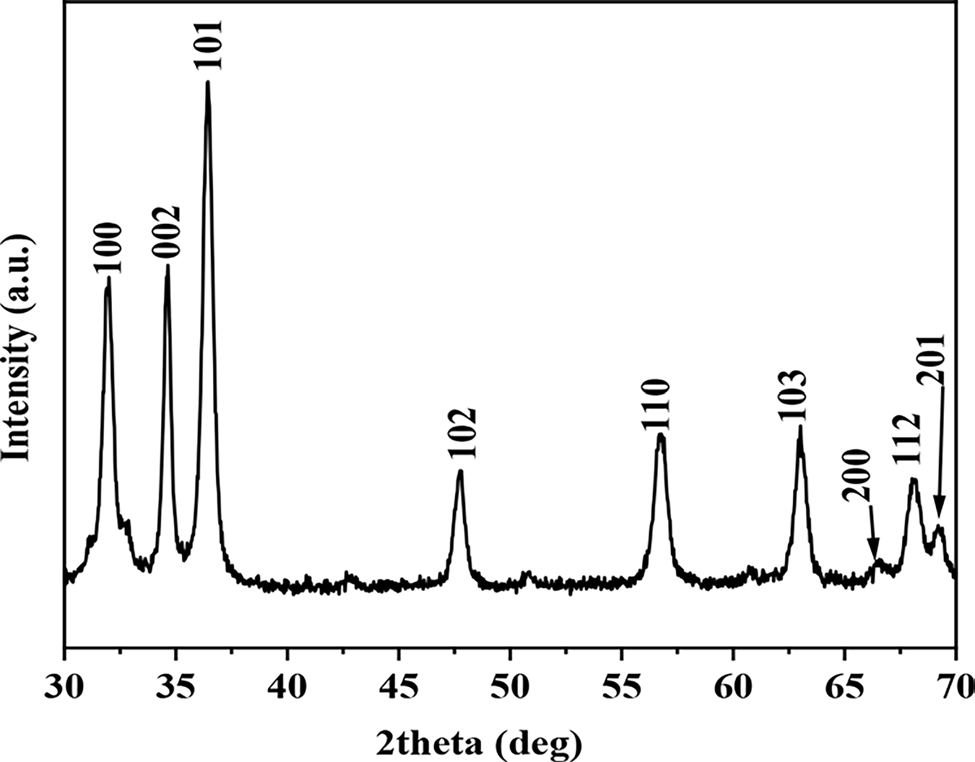
XRD Pattern of synthesized ZnO NPs using aqueous *Enterobacter sp*

The biosynthesized ZnOs NP by *E. sp.* was established via the UV–visible absorption spectra at the wavelength range of 300 to 360 nm which is the typical wavelength range of ZnO NPs. The biosynthesized ZnO NPs displayed a Surface Plasmon Resonance (SPR) band at 375 nm as shown in Fig. (1). While, in Fig. (2), an EDX study was carried out. Utilizing an aqueous extract of *E. sp*., the number of Bragg reflections for ZnO NPs utilizing this extract is 2θ 32.2 34.4, 36.5, 47.59, 56.65, 63.94, 67.46, 68.00, and 69.09, corresponding to the reflection from (100), (002), (101), (1 02), (1 0 3), (200), (112) to (2 0 1), respectively. The current finding demonstrated strong agreement with earlier findings that unveiled ZnO NPs’ hexagonal wurtzite structure.

**Fig. 3:**
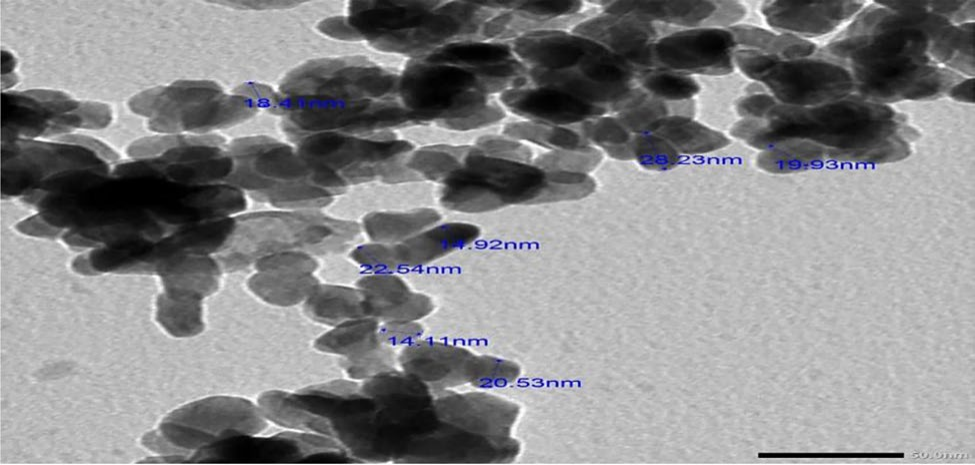
TEM image of synthesized ZnO NPs using *Enterobacter sp*

**Fig. 4:**
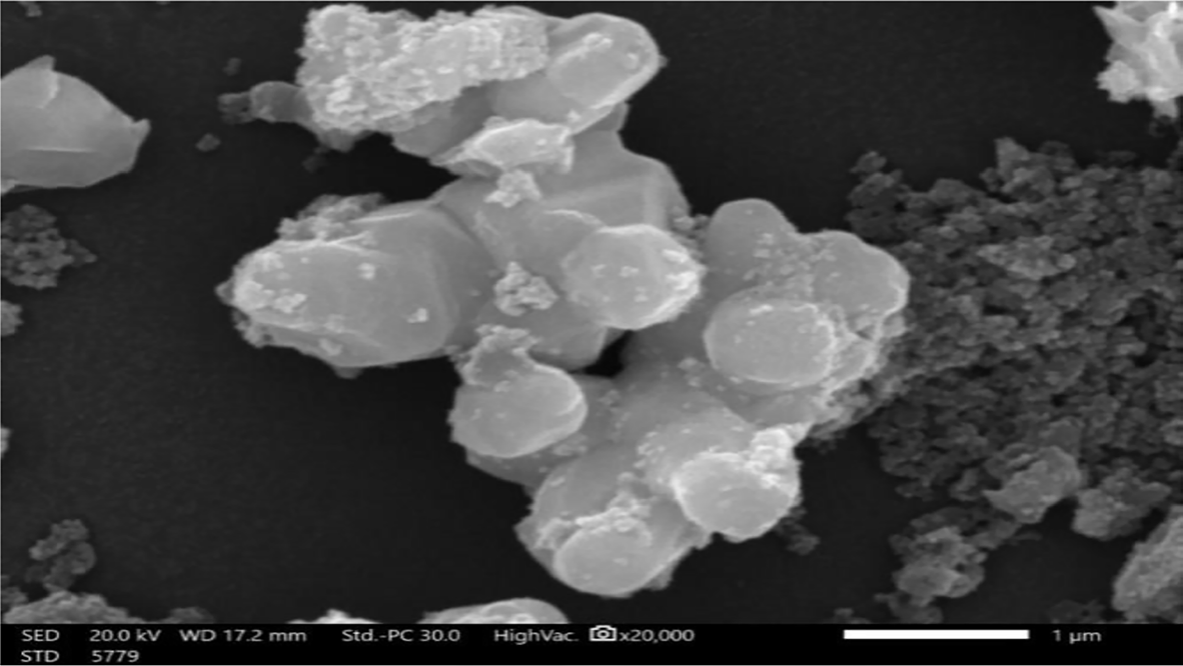
SEM image of green-synthesized ZnO NPs using *Enterobacter sp*

The size and shape of the produced ZnO NPs assessed using transmission and scanning electron microscopy (TEM) measurements; the outcome is shown in Fig. (3). The size range of 14.92 to 22.54 nm and the aggregated hexagonal structure of ZnO NPs displayed in Fig. (3). Many of the particles under analysis had homogeneous sizes. The green ZnO NPs that were produced had a mean value of 18.4 nm. The current discovery demonstrated the bacterial extract’s potential as a capping and reducing agent. The isothermal evaporation approach exhibited in Fig. (4) resulted in the growth of large-size single crystals measuring 30 × 17.2 × 8 mm, which were clear and nicely faceted when examined with a scanning electronic microscope (SEM).

**Fig. 5:**
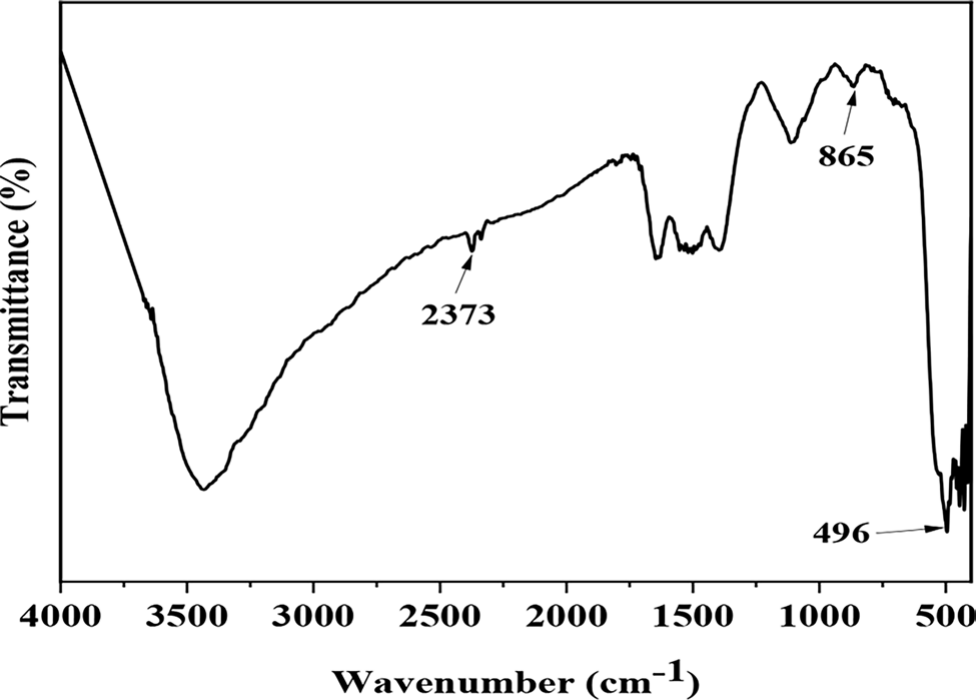
FTIR spectrum of synthesized ZnO NPs using *Enterobacter sp*

FTIR analysis was performed for the ZnO NPs recognition of the biomolecules responsible for the biosynthesis of ZnO NPs to evaluate the existence or absence of the different vibrational techniques for ZnO NPs. The accessible functional group of the phytochemical substances implicated in the stability and reduction of ZnO NPs may be found via FTIR analysis. Figure (5). The functional group of the bacterial supernatants was identified by the *E. sp*. extract’s FTIR spectra (Fig. 5). The bacterial supernatant showed Different peaks at 3420, 2373, 1715, 1685, 865, and 496 cm^− 1^. The absorption band at 3420 cm^− 1^ with novel peaks at established fatty acids, protein, and carbohydrate molecules because of increased secondary metabolites in the bacterial supernatant. Various overlapping bands were detected from 1715 to 865 cm^− 1^ region revealed carbonyl groups and the band at 1658 cm^− 1^ revealed carbonyl amides. In ZnO NPs, an intrinsic absorption peak was observed at 496 cm ^− 1^, which revealed the presence of stretching mode and OH group.

ZnO NPs mediated by *E. sp*. were tested for their antifungal and antibacterial properties. The *microorganisms utilized were S. typhimurium, K. pneumoniae, E. coli, and C. albicans*. The outcomes were contrasted with those of the widely used, commercially accessible medication tetracycline. When compared with fungal strains, the produced ZnO NPs have very favorable outcomes against antibacterial strains. Most of the precipitating ZnO NPs had antibacterial and antifungal action, albeit to differing degrees, according to data in Table 1. This discrepancy might result from the nanoparticles’ varied interactions with the many creatures under test. Additionally, it was discovered that the ZnO NPs’ minimum inhibitory concentrations (MICs) for *E. Coli, S. Typhimurium, K. pneumoniae*, and *C. albicans* were, 0.4, 0.9, 1.0, and 1.5 mg/mL respectively, as shown in (Table 1).

**Table 1:** Antimicrobial activity and minimum inhibition concentration of the synthesized ZnO nanoparticles on different microorganisms compared with standard antibiotic

Microbial strains	Diameter of Inhibition zone (mm)	Stander antibiotics Tetracycline (zone mm)	Minimum inhibition concentration (MIC) (mg/ml)
*Escherichia coli*	1.5 ± 0.32^D^	1.3	0.4
*Salmonella typhimurium*	2.6 ± 0.21^A^	2.7	0.9
*Klebsiella pneumoniae*	1.9 ± 0.10^C^	1.8	0.1
*Candida albicans*	2.3 ± 0.22^B^	2.1	1.5

The same letters in the column indicate no significant difference (*p* < 0.05) as analyzed by the Duncan test. Each value is the average of 3 replicates ± SD.

Results in Table 2 show the effects of Cu, bacterial suspension, and/or different concentrations of ZnO NPs on the germination percentage (GP%), of *Vicia faba*. shoot height, root depth, the fresh mass (FM) of the shoot, and root, dry mass (DM) of the shoot and leaf area of the faba bean seedling. Our results indicate that copper stress (100 mM CuSO_4_) significantly decreased the germination percentage (GP%) by 66.7% and declined all measured growth parameters of faba bean seedlings compared with the control. Application of the bacterial suspension and different concentrations of ZnO NPs increased all measured growth parameters in all treated faba bean seedlings compared with the control (Table 2). Moreover, different concentrations of ZnO NPs reduced the harmful effects of copper stress and increased all growth parameters in Cu-stressed seedlings as shown in Table 2 and Fig S1. Furthermore, the data revealed that the highest concentration of ZnO NPs (1000 mgL^− 1^) was the effective concentration among all treatments compared with control and Cu stress (Table 2).

**Table 2:** Effect of different concentration of ZnONPs and bacterial suspension on germination percentage (GP%), shoot height, root depth, shoot fresh mass (Shoot FM), root fresh mass (Root FM), shoot dry mass (Shoot DM), root dry mass (Root DM) and leaf area of 21-day-old cu-stressed *Vicia faba* (L.) seedlings

Treatments	GP (%)	Shoot height (cm plant^− 1^)	Root depth (cm plant^− 1^)	Shoot FM (g organ^− 1^)	Root FM (g organ^− 1^)	Shoot DM (g organ^− 1^)	Root DM (g organ^− 1^)	Leaf area (cm^2^ plant^− 1^)
Control	100 ± 0.00^A^	16.3 ± 0.61^B^	20.2 ± 0.72^A^	2.9 ± 0.59^C^	1.4 ± 0.03^B^	0.82 ± 0.03^B^	0.43 ± 0.04^C^	17.9 ± 0.43^B^
Bacterial Sus.	86.6 ± 4.7^B^	15.3 ± 0.42^B^	17.8 ± 0.03^B^	2.4 ± 0.08^C^	1.4 ± 0.21^B^	0.80 ± 0.06^B^	0.52 ± 0.04^B^	15.6 ± 0.33^C^
ZnO NPs 250 mgL^− 1^	100 ± 0.00^A^	17.9 ± 0.63^B^	20.4 ± 0.44^A^	3.7 ± 0.07^B^	2.0 ± 0.41^A^	1.00 ± 0.09^A^	0.58 ± 0.04^B^	19.5 ± 0.25^A^
ZnO NPs 500 mgL^− 1^	100 ± 0.00^A^	18.0 ± 0.34^A^	20.8 ± 0.53^A^	3.7 ± 0.06^B^	2.1 ± 0.02^A^	1.05 ± 0.05^A^	0.69 ± 0.04^A^	19.8 ± 0.76^A^
ZnO NPs 1000 mgL^− 1^	100 ± 0.00^A^	19.7 ± 0.83^A^	21.8 ± 0.32^A^	4.8 ± 0.09^A^	2.4 ± 0.13^A^	1.06 ± 0.03^A^	0.79 ± 0.04^A^	20.7 ± 0.86^A^
Cu100mM	33.3 ± 4.72^E^	10.0 ± 0.42^E^	10.6 ± 0.95^F^	1.2 ± 0.07^D^	0.54 ± 0.02^E^	0.041 ± 0.05^E^	0.13 ± 0.06^F^	10.8 ± 0.23^F^
Bacterial Sus.+ Cu100 mM	60.0 ± 8.12^D^	10.4 ± 0.44^E^	12.3 ± 0.53^E^	2.3 ± 0.09^C^	0.69 ± 0.02^DE^	0.051 ± 0.06^D^	0.22 ± 0.01^E^	12.5 ± 0.36^E^
ZnO NPs 250 mgL^− 1^+Cu100 mM	73.3 ± 4.71^C^	11.7 ± 0.45^D^	13.6 ± 1.05^D^	2.1 ± 0.08^C^	0.72 ± 0.03^D^	0.06 ± 0.013^C^	0.27 ± 0.03^E^	14.0 ± 0.14^D^
ZnO NPs 500 mgL^− 1^ +Cu100mM	76.6 ± 4.74^C^	13.1 ± 0.07^C^	14.4 ± 0.25^CD^	2.5 ± 0.40^C^	0.93 ± 0.02^C^	0.061 ± 0.06^C^	0.33 ± 0.03^D^	14.3 ± 1.17^D^
ZnO NPs 1000 mgL^− 1^+Cu100 mM	86.6 ± 4.75^B^	13.8 ± 0.48^C^	15.5 ± 0.36^C^	2.5 ± 0.45^C^	1.2 ± 0.23^B^	0.069 ± 0.07^C^	0.38 ± 0.06^D^	15.7 ± 0.95^C^

The same letters in each column indicate no significant difference (*p* < 0.05) as analyzed by the Duncan test. Each value is the average of 3 replicates ± SD.

The data in Table 3 reveals that treatment with Cu led to a noticeable reduction in the photochemical activity (Fv/Fm), and photosynthetic pigments (Chl a and Chl b) with percentages of decrease of 43%, 67%, and 53%, respectively compared with the control. On the contrary, the Carotenoid ratio exhibited a highly significant enhancement with copper stress treatment represented by 1.3-fold compared with the control. Conversely, the Cu-induced reductions in the photosynthetic pigments and activity recovered with the bacterial suspension treatments and different concentrations of ZnO NPs. In this regard, they caused a significant increase in the content of Chl a, Chl b, and Fv/Fm compared to the stress treatment; where the results indicated that the highest concentration of ZnO NPs (1000 mgL^− 1^) had the highest pronounced increase respectively relative to Cu treatment. Moreover, the carotenoids were enhanced by different concentrations of Zn ONPs, whereas (1000 mgL^− 1^ ZnO NPs) treatment caused the highest impact with a decrease of the carotenoid percentage of 39% relative to Cu treatment (Table 3).

**Table 3:** Effect of different concentrations of ZnO NPs and bacterial suspension on Photosynthetic activity (Fv/Fm), and contents of Chlorophyll a (Chl a), Chlorophyll b (Chl b), Carotenoids (Carot.), Total soluble proteins (TSP), Total soluble carbohydrates (TSC), Total phenolic (T. Ph) and Total Flavonoid (T. Flav) of 21-day-old cu-stressed *Vicia faba* (L.) seedlings

Treatments	Fv/Fm	Chl a (mg g^− 1^ FM)	Chl b (mg g^− 1^ FM)	Carot. (mg g^− 1^ FM)	TSP (mg g^− 1^ DM)	TSC (mg g^− 1^ DM)	T.Ph (mg g^− 1^ DM)	T.Flav (mg g^− 1^ DM)
Control	0.62 ± 0.01^C^	6.29 ± 0.13^B^	3.53 ± 0.28^B^	0.298 ± 0.01^E^	34.20 ± 2.4^E^	3.33 ± 0.47^E^	14.86 ± 0.40^E^	3.63 ± 0.76^D^
Bacterial Sus.	0.69 ± 0.01^BC^	7.15 ± 0.43^A^	3.48 ± 0.05^B^	0.327 ± 0.02^D^	33.07 ± 1.7^E^	4.39 ± 0.35^D^	13.06 ± 1.75^F^	3.44 ± 0.83^D^
ZnO NPs 250 mgL^− 1^	0.72 ± 0.01^B^	7.43 ± 0.08^A^	3.71 ± 0.15^B^	0.314 ± 0.06^D^	35.46 ± 2.5^D^	4.46 ± 0.44^D^	15.46 ± 1.51^E^	3.70 ± 0.83^D^
ZnO NPs 500 mgL^− 1^	0.76 ± 0.03^AB^	7.48 ± 0.19^A^	4.46 ± 0.44^A^	0.398 ± 0.01^D^	37.46 ± 0.4^C^	4.66 ± 0.40^D^	16.46 ± 1.16^D^	4.46 ± 0.85^C^
ZnO NPs 1000 mgL^− 1^	0.82 ± 0.01^A^	7.78 ± 0.39^A^	4.99 ± 0.73^A^	0.398 ± 0.01^D^	37.46 ± 2.0^C^	3.73 ± 0.82^E^	16.13 ± 1.19^D^	4.99 ± 0.91^C^
Cu100mM	0.35 ± 0.03^F^	2.03 ± 0.63^F^	1.63 ± 0.23^E^	0.694 ± 0.01^A^	54.63 ± 1.3^A^	9.37 ± 0.28^A^	24.63 ± 1.16^A^	9.63 ± 1.01^A^
Bacterial Sus. +Cu100 mM	0.41 ± 0.01^E^	3.33 ± 0.47^E^	2.23 ± 0.13^D^	0.567 ± 0.01^B^	43.06 ± 1.7^B^	6.43 ± 0.08^B^	20.70 ± 0.08^B^	5.23 ± 0.74^B^
ZnO NPs 250 mgL^− 1^ + Cu100 mM	0.43 ± 0.04^E^	4.39 ± 0.35^D^	2.43 ± 0.06^D^	0.430 ± 0.01^C^	42.03 ± 1.5^B^	5.77 ± 0.48^C^	20.23 ± 0.16^B^	5.10 ± 0.79^B^
ZnO NPs 500 mgL^− 1^ +Cu100 mM	0.47 ± 0.02^DE^	4.46 ± 0.44^D^	2.63 ± 0.23^D^	0.425 ± 0.02^C^	36.40 ± 1.4^D^	4.77 ± 0.94^D^	20.03 ± 0.40^B^	4.97 ± 0.81^C^
ZnO NPs 1000 mgL^− 1^ +Cu100 mM	0.50 ± 0.01^D^	5.33 ± 0.40^C^	3.06 ± 0.12^C^	0.422 ± 0.02^C^	35.36 ± 1.0^D^	4.11 ± 0.09^D^	19.23 ± 0.73^C^	3.73 ± 0.82^D^

The same letters in each column indicate no significant difference (*p* < 0.05) as analyzed by the Duncan test. Each value is the average of 3 replicates ± SD

It is apparent from the results in Table 3 that Cu stress significantly elevated the total soluble carbohydrates (TSC), total soluble proteins (TSP), total phenolic (T. Ph), and total flavonoid (T. Flav) of the seedling’s shoots by 1.8-fold, 59%, 65%, and 1.6-fold, respectively compared to the control. On the other hand, the treatment with bacterial suspension or ZnO NPs recovered the toxic impacts of Cu on the TSC, TSP, T. Ph, and T. Flav of stressed seedlings. Among these treatments, the ZnO NPs significantly reduced the contents of measured compounds than the Cu treatment to reach near the control value whereas the ZnO NPs (1000 mg L^− 1^) treatment caused the most significant effect reaching even over the control (Table 3).

The effects of ZnO NPs, bacterial suspension, and/ or Cu on (MDA), H_2_O_2_, and the Antioxidant enzyme activities in shoots of faba bean seedlings are shown in Table 4. Cu induced a significant increase in the content of MDA and H_2_O_2_ and the activity of SOD, CAT, and POX in comparison to the control. Indicating an alleviating capacity of Cu stress, bacterial suspension, and different concentrations of ZnO NPs inhibited the Cu-stimulated antioxidant enzymes. ZnO NPs significantly diminished the content of MDA and H_2_O_2_ and the activity of SOD, CAT, and POX relative to the Cu stress treatment and became lower even than the control whereas ZnO NPs (1000 mgL^− 1^) caused a significant reduction in the content of MDA and H_2_O_2_ also, the activities of SOD, CAT, and POX compared to stress treatment and reached approximately near the control values (Table 4).

**Table 4:** Effect of different concentrations of ZnONPs and bacterial suspension on the activity of antioxidant enzymes (Catalase; CAT, Superoxide dismutase; SOD, Peroxidase; POX) and the content of MDA and H_2_O_2_ of 21-day-old cu-stressed *Vicia faba* (L.) seedlings

Treatments	CAT (U min^− 1^ mg^− 1^ protein)	SOD (U min^− 1^ mg^− 1^ protein)	POX (U min^− 1^ mg^− 1^ protein)	MDA (µmol g^− 1^ FM)	H_2_O_2_ (µmol g^− 1^ FM)
Control	8.44 ± 0.47^C^	0.083 ± 0.004^D^	3.38 ± 0.004^D^	15.33 ± 0.18^D^	0.153 ± 0.001^E^
Bacterial Sus.	9.05 ± 0.46^B^	0.064 ± 0.011^F^	3.38 ± 0.004^D^	13.06 ± 0.09^E^	0.129 ± 0.01^G^
ZnO NPs 250 mgL^− 1^	8.86 ± 0.54^C^	0.073 ± 0.015^E^	3.63 ± 0.208^D^	13.09 ± 1.35^E^	0.145 ± 0.009^F^
ZnO NPs 500 mgL^− 1^	8.50 ± 0.42^C^	0.073 ± 0.016^E^	3.44 ± 0.241^D^	12.90 ± 1.83^F^	0.139 ± 0.02^F^
ZnO NPs 1000 mgL^− 1^	8.40 ± 0.08^C^	0.070 ± 0.016^E^	3.70 ± 0.360^D^	11.01 ± 0.81^G^	0.110 ± 0.008^G^
Cu100mM	11.7 ± 0.47^A^	0.143 ± 0.014^A^	8.68 ± 0.470^A^	25.79 ± 0.63^A^	0.257 ± 0.006^A^
Bacterial Sus. +Cu100 mM	9.6 ± 0.57^B^	0.138 ± 0.008^A^	5.13 ± 0.004^B^	18.86 ± 0.18^B^	0.227 ± 0.04^B^
ZnO NPs 250 mgL^− 1^ +Cu100 mM	8.9 ± 0.004^C^	0.104 ± 0.008^B^	5.77 ± 0.481^B^	17.09 ± 1.23^C^	0.229 ± 0.04^B^
ZnO NPs 500 mgL^− 1^ +Cu100 mM	8.8 ± 0.16^C^	0.099 ± 0.004^C^	4.77 ± 0.944^C^	17.04 ± 0.92^C^	0.190 ± 0.009^C^
ZnO NPs 1000 mgL^− 1^ +Cu100 mM	8.6 ± 0.47^C^	0.086 ± 0.004^D^	4.11 ± 0.093^C^	16.03 ± 0.08^D^	0.179 ± 0.013^D^

The same letters in each column indicate no significant difference (*p* < 0.05) as analyzed by the Duncan test. Each value is the average of 3 replicates ± SD.

**Fig. 6:**
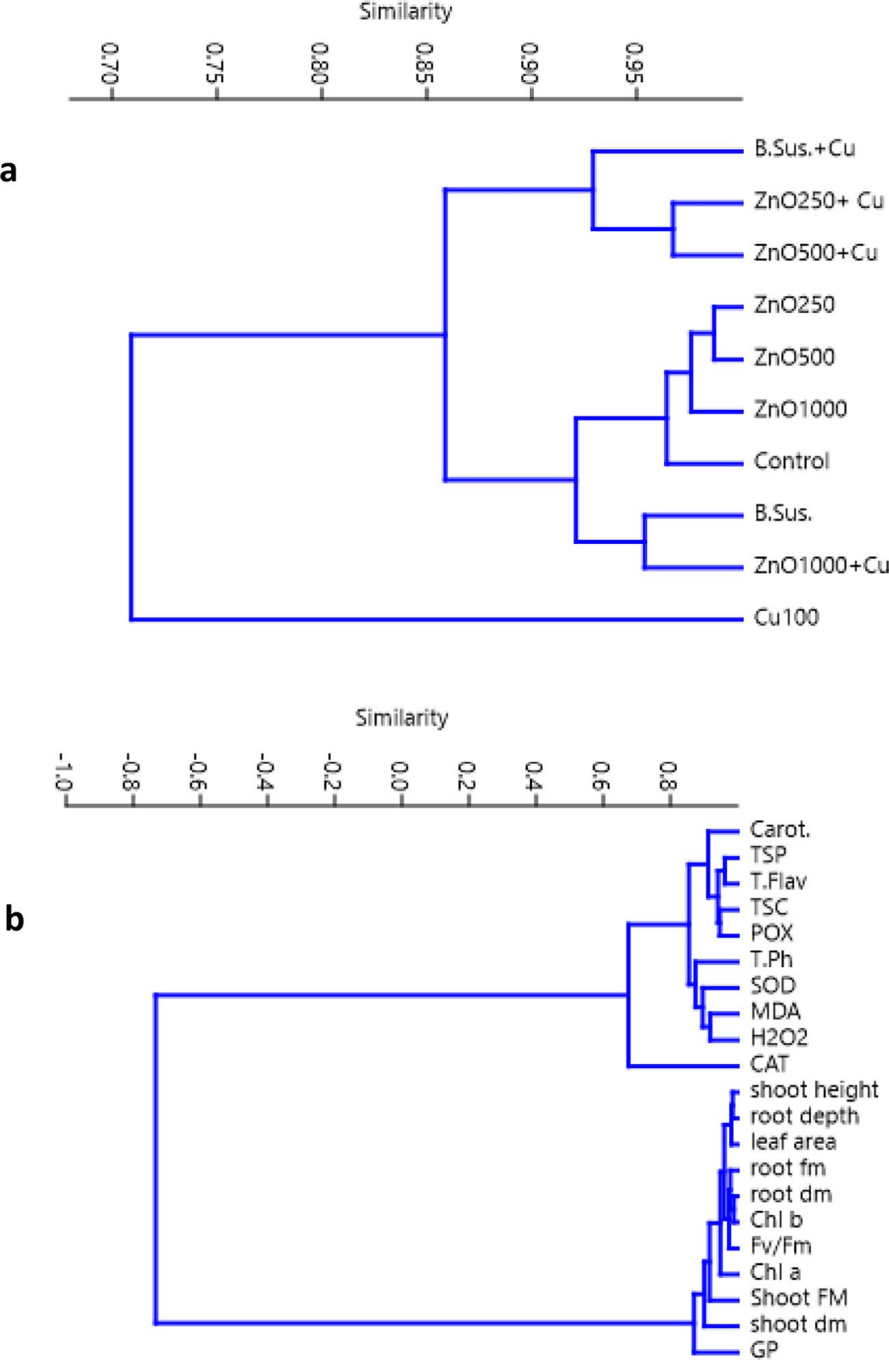
Multivariate cluster analysis of 21-day-old cu-stressed *Vicia faba* (L.) seedlings responses and the study treatments (**a**) and morpho-physiological traits (**b**). Abbreviations; B.Sus. = Bacterial suspension, Cu = 100mM CuSO_4_, ZnO 250 = ZnO NPs (250 mgL^− 1^), ZnO 500 = ZnO NPs (500 mgL^− 1^), ZnO 1000 = ZnO NPs (1000 mgL^− 1^). The tested variables included germination percentage (GP), shoot height, root depth, shoot fresh mass (Shoot FM), root fresh mass (root FM), shoot dry mass (Shoot DM), root dry mass (Root DM), leaf area, maximum PSII (Fv/Fm), Chl a, Chl b, carotenoids (Carot.), total soluble proteins (TSP), total soluble carbohydrates (TSC), total phenolic (T. Ph), total flavonoid (T. Flav), catalase (CAT), superoxide dismutase (SOD), peroxidase (POX), malondialdehyde (MDA) and hydrogen peroxidase (H_2_O_2_)

The interpretive methods for showing the level of similarity among more than two treatments or features are improved by multivariate classification (cluster analysis). Then, using the data gathered, it was feasible to identify any parallels or variations in seedlings’ morpho-physiological characteristics and their responses to the treatments (Fig. 6). Under the Cu stress, plant responses to the treatments showed that they all followed the same trend (Fig. 6b). While this was going on, the cluster (Fig. 6a) demonstrated substantial correlations between the control and Cu stress (with both bacterial suspension and different concentrations of ZnO NPs).

The interpretation procedures for determining the extent of similarities among multiple treatments or features are improved by the cluster analysis as well. Data shown in Fig. (6a) showed that plant responses to treatments were more similar between (control and different concentrations of ZnO NPs) indicating that this subcluster is the most significant effective group in enhancing all control seedlings and ameliorating the harmful effect of Cu stress, while, the second subclusters containing the bacterial suspension treatment and low concentrations of ZnO NPs (250 and 500 mgL^− 1^) also, had a potent to alleviated the Cu stress in *V. faba* (L.) individuals but their effects still lower that the highest concentration of ZnO NPs (1000 mgL^− 1^) (Fig. 6a).

The measured plant attributes showed substantial to highly positive significant relationships (Fig. 6b). Plant characteristics were divided into two sub-clusters. There was a high significant negative correlation between the first sub-cluster parameters including (Carot., SOD, POX, CAT, H_2_O_2_, T. Flav, T. Ph, MDA, TSC, and TSP), and the second one (germination percentage, shoot height, root depth, shoot fresh mass, root fresh mass, shoot dry mass, root dry mass, leaf area, maximum PSII (Fv/Fm), Chl a, Chl b, ) therefore, a substantial connection with these clusters was found (r^2^ = 0.6** to 1.0***) (Fig. 6b).

**Fig. 7:**
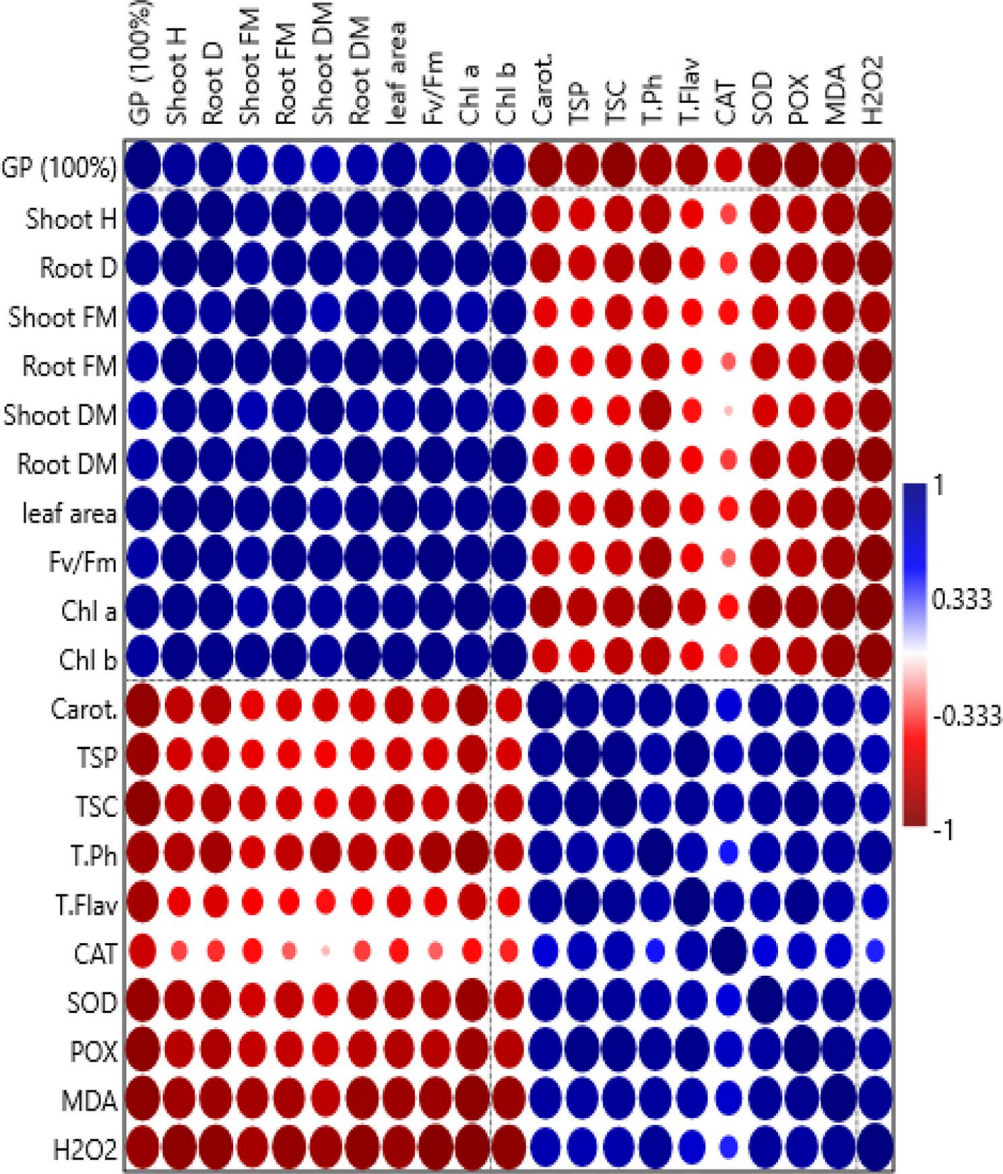
Heat map of person correlation analysis between all measured morphological and physiological parameters {(germination percentage (GP 100%), shoot height (shoot H), root depth (root D), shoot fresh mass (Shoot FM), root fresh mass (root FM), shoot dry mass (Shoot DM), root dry mass (Root DM), leaf area, maximum PSII (Fv/Fm), Chl a, Chl b, carotenoids (Carot.), total soluble proteins (TSP), total soluble carbohydrates (TSC), total phenolic (T. Ph), total flavonoid (T. Flav), catalase (CAT), superoxide dismutase (SOD), peroxidase (POX), malondialdehyde (MDA) and hydrogen peroxidase (H_2_O_2_)} of 21-day-old cu-stressed *Vicia faba* (L.) in response to different concentrations of ZnO NPs (250, 500 and 1000 mg/L^− 1^)

The correlation between the parameters obtained in the copper-stressed *V. faba* seedlings treated with bacterial suspension and/or different concentrations of ZnO NPs, Pearson’s correlation analysis (heat map) was created as shown in Fig. (7) of the variable traits. Pearson correlation coefficient (r) values were expressed from − 1/1 to 1, where − 1/1 denotes a perfect negative relationship, 1 denotes a perfect positive relationship, and 0 denotes no relationship at all between the variables under study (Fig. 7). The results showed a positive association between GP, shoot height, Root depth, Shoot FM, Shoot DM, Root FM, Root DM, and Leaf area in addition to, Chl a, Chl b, and Fv/Fm whereas the values of Pearson correlation coefficient ranging from (0.332 to 1) at (p **<** 0.05). There was an inverse relationship with Carotenoids, TSC, TSP, T. Ph, T. Flav, H_2_O_2_, MDA, and the activity of SOD, CAT, and POX whereas, the values of Pearson correlation coefficient ranged from (-0.333 to -1) at (p **<** 0.05) (Fig. 7).

## Discussion

The use of microbes in creating nanoparticles (NPs) has drawn a lot of interest recently as an alternative to chemical and physical processes. It was done to create ZnO NPs using *E. sp.* culture supernatants. One milligram of zinc ions was added to the cell filtrate of the examined bacterial strain. The development of colloidal zinc nanoparticles was indicated by the white material that appeared in the reaction vessels. It was suggested that biological substances produced into the supernatant by the bacteria and functional groups like protein and carboxylate that are present in the bacterial cell are what cause the reduction of Zn^2+^ to ZnO NPs [52].

An innovative method for characterizing ZnO NPs is UV-vis absorption spectroscopy. ZnO’s UV-vis spectrum has a distinctive peak at 375 nm, which is indicative of ZnO NPs and validates their creation. Additionally, the blue shift showed that the produced ZnO NPs had higher blue shift absorption than the bulk ZnO. This difference might be due to both a significant reduction in particle size and the large excitation binding energy of the ZnO NPs at ambient temperature. Absorption spectroscopy has long been used to confirm that the band gap increases with decreasing particle size. The results were comparable to those of earlier investigations by [52] who reported a similar absorption peak of the ZnO NPs that were biosynthesized.

To ascertain the crystalline structure and elemental content of the biosynthesized ZnO NPs, XRD analysis was performed on the ZnO NPs. Several Bragg reflections for ZnO NPs using an aqueous extract of *E. sp.* were found during the ZnO NPs element characterization. These reflections appear at 32.2, 34.4, 36.5, 47.59, 56.65, 63.94, 67.46, 68.00, and 69.09, respectively, and correspond to the refection from (100), (002), (101), (1 02), (11 0), (200), (112) to (2 0 1), respectively. The current discovery demonstrated strong agreement with earlier findings that demonstrated ZnO NPs’ hexagonal wurtzite structure [52].

TEM was used to examine the size and form of the green-produced ZnO NPs. The results unmistakably demonstrate the NPs’ hexagonal structure and size range of 14.92 to 22.54 nm. Most of the particles under analysis had homogeneous sizes. The green ZnO NPs that were produced had a mean value of 18.4 nm. The current discovery demonstrated seed extract’s potential as a capping and reducing agent. The hexagonal and spherical NP structures matched earlier findings [52, 53].

The biomolecules responsible for the biosynthesis of ZnO NPs identified via FTIR analysis. The accessible functional group from the phytochemical components implicated in the stability and reduction of ZnO NPs may be found via FTIR analysis. The functional group of the bacterial supernatants identified by the *E. sp.* extracts FTIR spectra. The bacterial supernatant showed Different peaks at 3420, 2373, 865, 1715,1685 and 496 cm -^1^. ZnO NPs participated in C = C and CAH bending, carbonyl groups, and carbonyl amides, as seen by the absorption band of new peaks at 2373, 1715,1685, 865 and 496 cm ^− 1^ The peak at 496 cm^− 1^ also showed the presence of stretching mode and OH group [54]. Zinc ions (Zn^2+^) were reduced to Zn^+ 1^ and then to zinc NPs by the electrons that the bacteria’s available functional group gave the bacterial supernatant functions as a stabilizing agent with negative functional groups. The ZnO vibrations were detected as a strong and sharp band at 496 cm^− 1^, while the weak vibration was detected as a peak at 865 cm^− 1^ [55]. Wave numbers less than 1000 cm^− 1^ are indicative of very strong absorption bands that are generally present in meal oxides [52]. An inherent absorption peak in ZnO NPs found at 496 cm ^− 1^, indicating the presence of an OH group and a stretching mode at higher wave numbers [52].

The powdered zinc nanoparticles produced by bacterial organisms exhibited varying inhibitory effects on distinct human pathogenic bacteria, including *S. Typhimurium, K. pneumoniae*, *E. coli*, and the yeast *C. albicans*, which resembled fungus and displayed distinct inhibition zones of varying sizes. According to the data, most of the precipitated zinc nanoparticles had variable degrees of antibacterial and antifungal activity. This discrepancy might result from the nanoparticles’ varied interactions with the species under test. Part of the process by which zinc ions suppress bacteria is understood [56]. The antibacterial action might include the buildup of ZnO NPs in the cytoplasm or outer membrane of bacterial cells, which would induce the release of Zn^2+^ and lead to the disintegration of bacterial cell membranes, damage to membrane proteins, and genetic instability, causing the death of bacterial cells [57, 58]. It was demonstrated that ZnO NPs, which had an average size of around 30 nm, killed cells by coming into close contact with the membrane’s phospholipid bilayer and damaging its integrity. The bactericidal effect of ZnO NPs inhibited by the addition of radical scavengers such as glutathione, vitamin E, and mannitol. This might indicate that the generation of ROS was essential for ZnO NPs’ antibacterial qualities. However, it did not appear that Zn^2+^ released from ZnO NP suspensions had an antimicrobial impact. The results of testing ZnO NPs against various isolates revealed that their minimum inhibitory concentrations (MICs) varied in terms of antibiotic resistance profiles, suggesting that ZnO NPs had strong antibacterial action. So, it is reasonable to assume that eluted Zn^2+^ from ZnO NPs contributes significantly to the antibacterial effect.

Among metals that are highly persistent in the environment are heavy metals, and copper is one of the micronutrients that is crucial for plant growth and development [59]. High concentrations of Cu are regarded as a stress factor and hurt several physiological processes that result in plant mortality, even though Cu plays important roles in many physiological processes [60, 61]. The reduced biomass and growth seen in response to Cu stress may be caused by excess Cu metal interfering with several physiological and biochemical processes that are necessary for healthy plant root cells in conjunction with the suppression of mitotic activity [62, 63]. As seen in Cu-treated *Phaseolus vulgaris* [64] and *Oryza sativa* [65], Cu-induced cell wall rigidity linked to the strong induction of cell wall peroxidase activity and increasing its rigidity may be the cause of such growth inhibition [66, 67].

The present findings demonstrated that applying Cu significantly reduced both the photosynthetic activity and the pigments involved in photosynthesis (chlorophyll a and chlorophyll b) and increased the content of carotenoids. Ali et al. [68] observed similar outcomes in cabbage (*Brassica oleracea*) because of impaired chloroplast formation. Because of Cu’s interaction with free sulfur groups at the enzyme’s active site, as well as Fe and Mg deficiencies, the resulting Cu-induced reduction in chlorophyll content may be caused by inhibition of enzymes involved in the biosynthesis of photosynthetic pigments, restricting the formation of 5-aminolevulinic acid and protochlorophyllide reductase activity [69], according to [70]. Carotenoids are known to improve the ability of chlorophylls to withstand peroxidation, hence Cu-induced oxidative stress may increase the amount of these compounds. Furthermore, the stronger antioxidative state of the plants under investigation is shown by a notable rise in the carotenoid content in the leaves of the copper-resistant variety of faba bean seedlings as well as high activity of SOD and CAT in these leaves [71].

The results of this study clearly showed that treating the *V. faba* shoots with Cu stress increased their levels of total soluble carbohydrates (TSC), total soluble proteins (TSP), total phenolic (T. Ph), and total flavonoid (T. Flav) significantly. According to [72] this rise may offer an adaptive mechanism that maintains a positive osmotic potential in the face of Cu toxicity. According to [71] enhanced synthesis of stress-related proteins may be the cause of the buildup of proteins under stress. The subsequent rise in the overall flavonoid content under Cu stress indicated that it has metal-chelating qualities and plays a function in neutralizing free radicals to scavenge reactive oxygen species (ROS) [71]. *Orthosiphon stamineus* showed a similar rise in flavonoid content following Cu stress, as reported by [73]. According to [74] phenolic compounds function as pro-oxidants under abiotic stress, which means they can indirectly influence the concentration of harmful oxygen radicals like superoxide anion production in living cells. They can also bind metal ions, inhibit enzymatic systems that produce free radical forms, and induce the expression of enzymes’ genes that inhibit oxidative stress.

Results of the current study indicated a highly significant increase in both MDA and H_2_O_2_ under Cu stress. Cell membranes are the primary sites of injury under Cu stress, where membrane destabilization may often be attributed to the peroxidation of membrane lipids [75] and an increase in electrolyte leakage and MDA, which is the oxidized product of membrane lipids’ polyunsaturated fatty acids [76, 77]. Furthermore, under Cu stress, the highly destructive ROS, such as H_2_O_2_, are either indirectly produced by disrupting regular metabolic pathways [78] or directly produced by the Fenton-Haber-Weiss reaction [79], which is regarded as one of the most potent triggers of the production of free radicals [80].

As a result, the disturbance of cell homeostasis was the cause of the excessive generation of reactive oxygen species (ROS). As a protective response to oxidative stress, Cu stress dramatically increased the activity of CAT, POX, and SOD which was consistent with the study results of [79] who reported an induction in CAT and POX activities due to increasing Cu concentration in *B. juncea*. By causing the buildup of lignin, which is one of the components of the secondary cell wall, the increase in POX activity in response to Cu stress directly contributes to Cu-mediated growth suppression [81]. One of the essential enzymes in the elimination of harmful peroxides, catalase is an oxidoreductase that breaks down H_2_O_2_ into water and molecular oxygen. It is mostly located in peroxisomes [82]. SOD stimulates the dispersion of superoxide radicals (O_2_^−^) radicals and protects cells against ROS, which convert O_2_^−^ to H_2_O_2_ and O_2_, then POX and CAT, and finally detoxify H_2_O_2_ [83]. Because the ability of nanoparticles to promote plant growth and stress tolerance depends on their physicochemical properties, zinc oxide nanoparticles (ZnO NPs) under copper stress increased the tolerance of *V. faba* seedlings [84]. The growth and functions of the photosynthetic apparatus, pigment contents, total soluble protein and carbohydrate accumulation, antioxidant activity, and the content of phenolics and flavonoids of the studied faba bean seedlings under control and stressed environments were all clearly affected by ZnO NPs, as demonstrated by our results. Auxins and gibberellin are produced due to ZnO NPs’ positive effects on germination, which encourage the breakdown of seed stores and boost seed vigor [85]. The enhancement of traits linked to the seed’s physiological quality is ascribed to the introduction of nanoparticles (NPs) inducing photosensitization reactions and photo-production of active oxygen, including superoxide and hydroxide anions. For germination to occur quickly, these interactions encourage water and oxygen imbibition as well as ion penetration [86].

Zinc is an essential micronutrient needed for dry matter accumulation and plant growth [22]. ZnO NPs have a high absorption rate and transfer, ensuring an adequate quantity of Zn for its utilization in plant growth and development [87]. Zn could enhance membrane stability and cell elongation and division leading to an increase in fresh and dry weight [88]. ZnO NPs were extremely successful in decreasing Cu toxic effects and causing the measured parameters for growth by interacting with active biomolecules and activating various biochemical pathways [89]. According to [90], suggested that ZnO NP treatment decreased Cu buildup, which in turn lessened its harmful effects and contributed to the restoration of biomass.

Previous research on the impact of ZnO NPs during germination and seedling growth has shown both beneficial and phytotoxic effects. The examined species’ genotype, size, concentration, and type of NP are the primary causes of response variability [91]. The treatment of ZnO NPs in this study had an impact on the root and shoot growth of faba bean seedlings. It noted that because the root growth index is so sensitive to stress, it is frequently utilized as a measure of phytotoxicity [84]. Since the radicle is the earliest construction of the seed embryo, it is the first tissue subjected to high ZnO NP concentrations [64]. Because of this, radicles showed the negative impacts more clearly than shoots did. This shows that shoot elongation was less vulnerable to the toxicity of ZnO NPs than root elongation, a difference in sensitivity that is explained by roots being directly exposed to ZnO NPs [92]. Because of this, radicles showed the negative impacts more clearly than shoots did. This shows that shoot elongation was less vulnerable to the toxicity of ZnO NPs than root elongation, a difference in sensitivity that is explained by roots being directly exposed to ZnO NPs [93]. Different concentrations of ZnO NPs or dissolved zinc ions interacting with root biochemistry and physiology could be the source of the difference in root growth. Variations in root length and the buildup of dry biomass were encouraged by this intervention. Our findings are consistent with studies on radish, lettuce, cucumbers, and Arabidopsis that have noted inhibition and variance in root growth [94, 95].

Two primary effects could be responsible for this inhibition: First, there is chemical toxicity that depends on the release of (toxic) ions, such as when nanoparticles pierce through and Zn2 + ions dissolve from ZnO; second, there is stress or stimulation brought on by the surface, size, or form of the particles [95]. High doses of toxicity are consistent with Shelford’s tolerance law. A decrease in plant biomass and root length may result from the toxicity of intra- or intercellular aggregation of nanoparticles absorbed by the plant from the media [96]. Accordingly, ZnO NP treatments had an impact on the morphology of the faba bean seedlings because they had an oxidative stress-causing phytotoxic effect that increased the activation of secondary metabolism in this tissue [97]. Since it is essential to the plant’s reaction to NP treatment, the relationship between ROS and secondary signaling messengers that result in transcriptional regulation of the secondary plant metabolism is considered [98].

ZnO NPs preserved the amount of chlorophyll under Cu toxicity, which may be related to Zn’s significant role as a micronutrient in the biochemical reactions necessary for chlorophyll synthesis [99]. ZnO NPs may also improve the activity of carbonic anhydrase and increase the efficiency of chemical energy production in photosynthetic systems [25]. Also [100], also showed that zinc has a favorable influence on photosynthetic activity and that zinc plays a significant role in the structure of the chloroplasts and in photosynthetic electron transfer when zinc oxide nanoparticles (ZnO NPs) to wheat that is stressed by salinity. Additionally, zinc reduces ABA and controls stomata activities by holding onto the K content of protective cells [101]. Under the influence of ZnO NPs, a rise in the photosynthetic activity (Fv/Fm), Chl a and Chl b ratios may suggest a modification in the stoichiometry of the light-harvesting complexes of photosystems I and II and, consequently, a shift in their relative activities [102]. By speeding up the electron flow from active reaction centers to the quinone pool, raising the quantum yield of PSII, decreasing the percentage of incoming light that is directed toward non-photochemical quenching, and applying excitation pressure to the cytochrome complex, the application of metal oxide nanoparticles under stress conditions increased the photosynthetic efficiency of plants [103].

In wheat plants, zinc nano priming doubled the number of active reaction centers per chlorophyll molecule, leading to improved absorption, effective excitation energy trapping, and electron transport [104]. ZnO NPs also lessen the likelihood of oxidative damage in stressed plants by reducing the amount of incoming light-induced excited electrons that proceed toward non-photochemical quenching [105]. As in the previous part, oxidative stress, which is characterized by cell shrinkage or a loss in leaf area, is indicated by decreases in pigment contents under heavy metal stress [106]. According to the data, adding ZnO NPs raised the chlorophyll concentration, which is equal to what the control plants had. At copper stress, ZnO NPs normalize the chlorophyll content to that of control plants. The well-known adaptation process in our work by the rise in carotenoid content in the leaves of faba bean seedlings developed from seeds treated with ZnO NPs following copper stress action [107]. Specifically, because of the physicochemical characteristics of their molecules, carotenoids are low-molecular antioxidants whose biosynthesis in leaves increases in response to stress, on the one hand, quench reactive oxygen species and, on the other, broaden the absorption spectrum of available light radiation for plants [70]. Total soluble carbohydrates (TSC) maintained after treatment with ZnO NPs, which may be related to its function in activating the enzymes involved in photosynthesis and chlorophyll biosynthesis [107], as well as the accumulation, regulation, and transformation of carbohydrates and sugars [108]. These results are consistent with the findings of [109] who used nano-iron and nano-zinc to boost TSC in salt-stressed *Moringa peregrina*.

Overall, a higher TSC is associated with a faster development rate; carbs serve as essential energy sources and the carbon skeletons that support organic molecules and storage elements [110]. When ZnO NPs utilized, the TSP increased significantly in comparison to the Cu stress treatment. These outcomes were consistent with those of [111], who found that adding ZnO NPs improved TSP and reduced negative effects caused by Pb and Cd in *Leucaena leucocephala* seedlings, which is an adaptive response to heavy metal stress. Zinc has been found to play a significant role in the structure of several enzymes and transcription factors that are involved in protein synthesis and regulation [109]; in the meantime, nanoparticles with a high surface-to-volume ratio are more reactive and may have more biochemical activity [112]. To reduce excess ROS and lessen the consequences of oxidative stress brought on by ZnO NPs, plants engage both enzymatic and non-enzymatic antioxidant defense systems. The primary defense mechanism is secondary plant metabolism activation, and phenolic chemical production is essential [113]. Phenolic compounds are important in the detoxification of reactive oxygen species (ROS) and their concentration can fluctuate greatly [114]. Phenolic compounds function as metal chelators when heavy metal stress occurs. On the other hand, because of their redox characteristics, they can directly remove molecular species of active oxygen and are crucial for the absorption and neutralization of free radicals, the extinction of singlet and triplet oxygen, and the breakdown of peroxides [115]. Thus, a rise in phenolic compounds which are potent ROS scavengers and capable of blocking enzymes that create free radicals is primarily responsible for the increases in antioxidant activity of plants treated with NPs [116].

Accordingly [116], revealed that ZnO NPs induced the formation of important secondary metabolites, including phenolic compounds in *B. nigra*. This is attributed to that zinc has an enhanced role in the shikimic acid cycle, which produces phenolic compounds that have a protective antioxidant impact [117]. Rather than chelating sites within the molecule, the general chelating capacity of phenolic compounds is connected to the strong nucleophilic character of the aromatic rings [118]. The balance of antioxidant defense systems in plants, however, is inhibited or modulated to avoid the oxidative stress that causes lipid peroxidation when the cellular concentration of ROS increases due to biotic and abiotic stresses [119]. This explains the maximum accumulation of phenolics and flavonoids in faba bean seedlings exposed to the highest concentration of ZnO NPs. According to reports, high Zn concentrations can raise the phenol content of Coriandrum sativum L. leaves and stems, but primarily in the radicles [120]. Radicles offer antioxidants to scavenge excessive ROS generation in addition to acting as chemical and physical barriers to biotic and abiotic stresses [121]. The trends in phenolic compounds and antioxidant activity are explained by the synergistic action of these compounds [122]. Comparable patterns have been in the antioxidant activity of *Stevia rebaudiana* Bertoni [123], *Salvia officinalis* L [124]. , and the radicle of *Brassica nigra* [125]. According to our findings, the activation of the antioxidative system by a notable increase in the *V. faba* enzymes catalase, peroxidase, and superoxide under copper stress and the impact of the binary composition of ZnO NPs linked to the potential contribution of nanoparticles to plant metabolism’s enzymatic reactions.

The ability of ZnO NPs to pass through epidermal cells and interact with the high-molecular organic components of cells due to their small size supports this theory. Simultaneously, the plasmodesmata have demonstrated the feasibility of nanoparticle transfer between cells [125]. In faba bean seedlings exposed to copper stress, we saw enhanced activity of the enzymes SOD, CAT, SOD, and POX, which is consistent with other investigations on various plants [126–128]. On the other hand, the administration of ZnO NPs under copper stress dramatically reduces the enzymatic activity, particularly SOD, CAT, and SOD; this may be because fewer ROS formed. This is also related to the fact that plants co-treated with ZnO NPs and copper have better uptake of zinc and other micronutrients, which is critical for reducing ROS formation and shielding plants from oxidative damage [117, 129]. ZnO NPs have a similar impact in lowering enzymatic activity (SOD, POD, and CAT) in nano primed Wheat plants [130]. Our research looked at how ZnO NPs prevented Cu-induced oxidative damage. The outcomes showed that in ZnO NPs challenged seedlings, MDA and H_2_O_2_ were reduced. Zinc can maintain and shield biomembranes from oxidative and peroxidative stress, preserve the integrity of the plasma membrane, alter its permeability, and modify free radicals and the processes associated with them through its antioxidant properties [131]. Additionally, zinc preferentially binds to the sulfhydryl groups of the membrane protein moiety [132].

## Conclusion

The biosynthesis of ZnO NPs exhibits biocompatibility and is efficient at penetrating cell walls due to their enhanced morphologies and ultra-small size. Zno NP’s effects of copper stress on *V. faba* are the overall outcome of a confluence of structural and functional elements that reduce physiological and plant development. ZnO NPs can effectively counteract changes and alter plant morphometric indexes. Furthermore, plants’ adaptation to copper stress under the ZnO NPs appeared in the ratio of chlorophyll a to b in the leaves. ZnO NPs improve the pro-oxidative/antioxidative balance and morphometric indicators and the activity of antioxidative enzymes. Reduced absorption and upward transport of Cu, and increased levels of promoting proteins, carbohydrates, phenolics, and flavonoids seemed the main drivers of their boosting effects. ZnO NPs have the potential in biomedical applications and to alleviate the harmful effects of heavy metals stress.

### Electronic supplementary material

Below is the link to the electronic supplementary material.

Supplementary Material 1

## Data Availability

All the data related to this work can be sourced from the corresponding authors.

## Abbreviations

ZnO NPs: Zinc oxide nanoparticles
Cu^+ 2^: Copper ions
TEM: Transmission electron microscopy
XRD: X-ray powder diffraction
FAIR: Fourier transformed infrared
UV-Vis: Ultraviolet-Visible spectroscopy
SEM: Scanning electron microscopy
MICs: Minimum inhibitory concentrations
*B. licheniformis*: *Bacillus licheniformis*
*LAB*: Probiotic lactic acid bacteria
*E. coli*: *Escherichia coli*
*S. aureus*: *Staphylococcus aureus*
MRS: Man, Rogosa, and Sharpe medium
Fv/Fm: The maximum efficiency of a photosystem PSII
Chl b: Chlorophyll b
Chl a: Chlorophyll a
Carot.: Carotenoids
SOD: Superoxide dismutase
CAT: Catalase
POX: Peroxidase
TSC: Total soluble carbohydrates
TSP: Total soluble proteins
T. Ph: Total phenolic
T. Flav: Total Flavonoid
GP: Germination percentage
DM: Dry mass
FM: Fresh mass
MDA: Malondialdehyde
H_2_O_2_: Hydrogen peroxide
ROS: Reactive oxygen species
O_2_^−^: Superoxide radical
