# Supplementary material for: Green-synthesized zinc oxide nanoparticles by Enterobacter sp.: unveiling characterization, antimicrobial potency, and alleviation of copper stress in Vicia faba (L.) plants
Source: BMC Plant Biol. 2024 May 30;24:474. doi: 10.1186/s12870-024-05150-0 (PMC11137959; doi:10.1186/s12870-024-05150-0)
Supplement: Supplementary file 1 — Supplementary Material 1 [file 12870_2024_5150_MOESM1_ESM.docx]

| 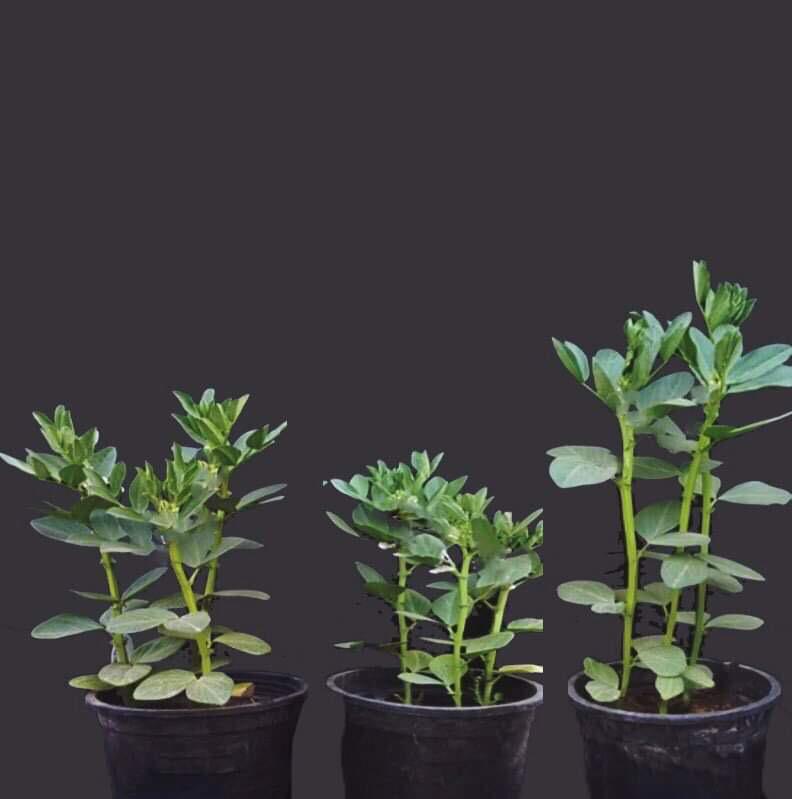  **Cu 100 mM**  **Control**  **Bacterial Suspension**  **+Cu 100 mM** | 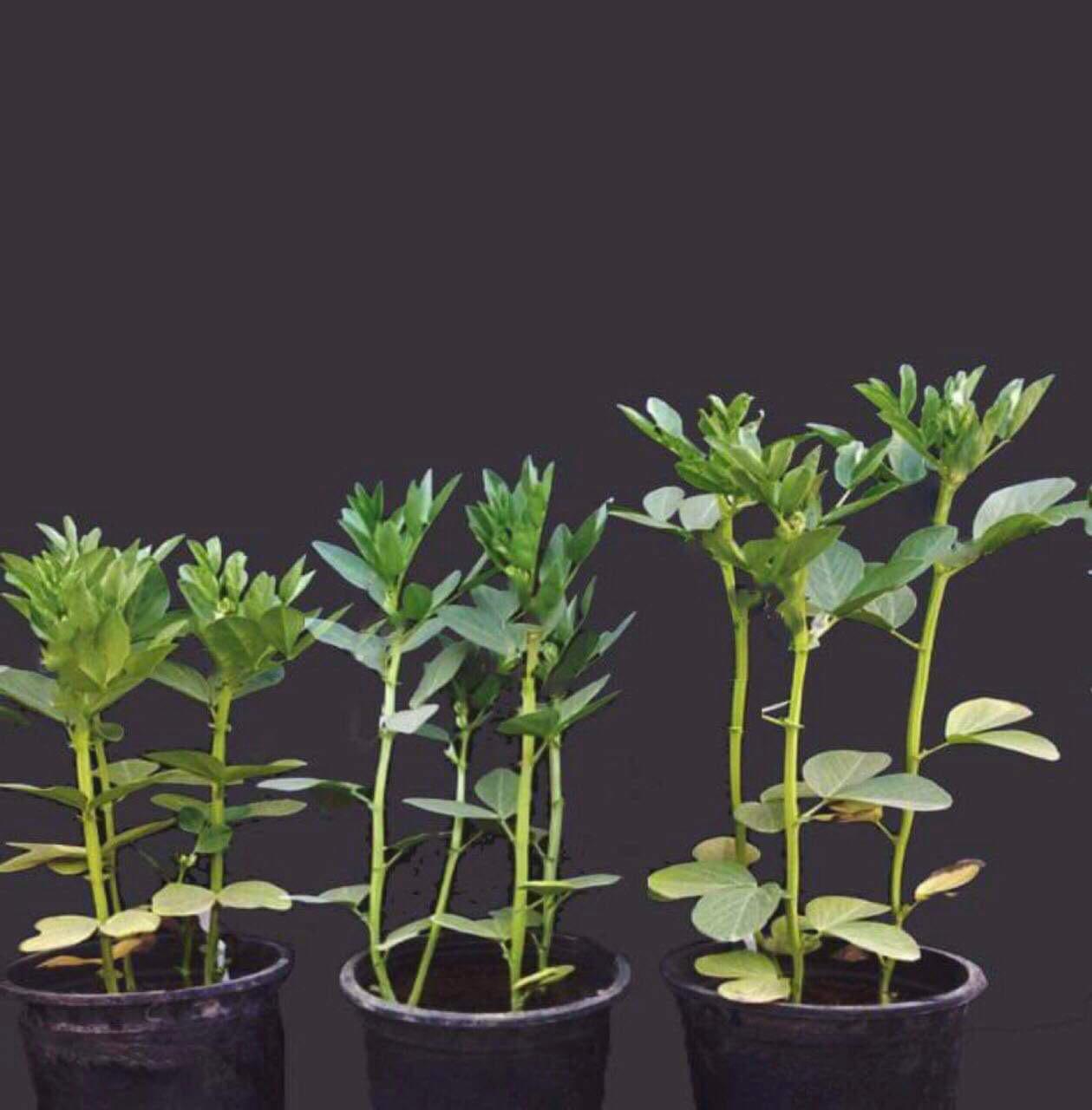  **ZnO NPs 1000 mg L^-1^ +Cu 100 mM**  **ZnO NPs 250 mg L^-1^ +Cu 100 mM**  **ZnO NPs 500 mg L^-1^ +Cu 100 mM** |
| --- | --- |

**(A) (B)**

Fig. S1: Effect of bacterial suspension (A) and different concentrations of ZnO NPs (B) on the morphological characters and growth of Cu-stressed *Vicia Faba* (L.) seedlings
